# The clinical and demographical characteristics of Turkish pediatric lymphedema patients: a multicenter study

**DOI:** 10.55730/1300-0144.5417

**Published:** 2022-03-26

**Authors:** Pınar BORMAN, Ayşegül BALCAN, Sibel EYİGÖR, Evrim COŞKUN, Figen AYHAN, Burcu DUYUR ÇAKIT, Seçil VURAL, Meltem VURAL, Esra Deniz Papatya ÇAKIR, Deniz ÇAĞDAŞ, Ayşegül YAMAN, Lale CERRAHOĞLU, Sevil CEYHAN DOĞAN

**Affiliations:** 1 Department of Physical Medicine and Rehabilitation, Ankara City Hospital, University of Health Sciences, Ankara Turkey; 2 Department of Physical Medicine and Rehabilitation, Faculty of Medicine, University of Hacettepe, Ankara, Turkey; 3 Department of Physical Medicine and Rehabilitation, Faculty of Medicine, University of Ege, İzmir, Turkey; 4 Department of Physical Medicine and Rehabilitation, Başak Çam Sakura City Hospital, University of Health Sciences, İstanbul, Turkey; 5 Department of Physical Medicine and Rehabilitation, Medicana Hospital, University of Atılım, Ankara, Turkey; 6 Department of Physical Medicine and Rehabilitation, Ankara Training and Research Hospital, University of Health Sciences, Ankara, Turkey; 7 Department of Physical Medicine and Rehabilitation, Bakırköy Sadi Konuk Training and Research Hospital, University of Health Sciences, İstanbul, Turkey; 8 Department of Pediatrics, Bakırköy Sadi Konuk Training and Research Hospital, University of Health Sciences, İstanbul, Turkey; 9 Department of Pediatrics, Faculty of Medicine, University of Hacettepe, Ankara, Turkey; 10 Department of Physical Medicine and Rehabilitation, Gülhane Education and Research Hospital, Ankara, Turkey; 11 Department of Physical Medicine and Rehabilitation, Faculty of Medicine, University of Celal Bayar, Manisa, Turkey; 12 Department of Physical Medicine and Rehabilitation, Faculty of Medicine, University of Cumhuriyet, Sivas, Turkey

**Keywords:** Child lymphedema, clinical, demographic, therapeutic, education

## Abstract

**Background/aim:**

Reducing lymphedema-associated burden and disability in the pediatric setting requires improved awareness and understanding clinical properties of the lymphedema. The aim of this study was to evaluate the clinical and demographic characteristics of patients with pediatric lymphedema presented to different lymphedema centers in Turkey.

**Materials and methods:**

The socio-demographic and clinical characteristics of the children including age, gender, presence of genetic syndromes, duration of edema, site and stage of lymphedema and the received therapies were determined. Parental and children education on self-management techniques were recorded.

**Results:**

A total of 122 children (female: 66, male: 56) with a mean age of 120.7 ± 71.2 months were included from 7 centers. Of them; 92% had primary, 8% had secondary lymphedema mostly due to infection and trauma. Lymphedema was part of a syndrome in 18% of the children. The most common site of involvement was the lower extremity, followed by upper extremity and genital involvement. Lymphedema was complicated in 17 % of children, mainly with a clinical picture of cellulitis, infection, and pain. The median duration of lymphedema was 41 (5–216) months. Although most of the children had stage 2 lymphedema, only 40% of them received treatment. The most commonly received treatment was compression therapy. No family or child was educated for self-care management before.

**Conclusion:**

In conclusion, pediatric lymphedema has a comparable gender distribution and usually involves the lower extremities. Although most of the children had advanced disease, more than half of the patients did not receive any treatment indicating the unmet need for management of lymphedema. The education of patients and/or children about self-management methods were lacking. We suggest educational activities for both families of children with lymphedema and health care providers, in order to facilitate early reference to lymphedema units and to receive prompt preventive and therapeutic approaches for this suffering condition.

## 1. Introduction

Lymphedema is a chronic progressive disease characterized by swelling of the body parts due to the insufficiency of the lymphatic system which can be seen in both adults and children [1–3]. Pediatric lymphedema is generally represented by developmental lymphatic vascular deficiency which can be either congenital or hereditary but it rarely occurs in children with an intact lymphatic system, due to secondary causes consisting infection, trauma, and other conditions [2–4]. Pediatric primary lymphedema may occur as a nonsyndromic inherited condition, or as a part of syndromic disorders comprising Turner, Noonan, Prader-Willi, Klippel-Trenaunay, vascular malformations and etc. [4–8]. In most cases lymphedema is present from birth, but may also develop later in some cases. The age of onset (congenital, pubertal), family history, site of swelling, associated conditions and dysmorphic features and underlying genetic causes are important in the differential diagnosis of pediatric lymphedema [2]. Diagnosis of pediatric lymphedema is based mainly on clinical findings but physicians have to take detailed anamnesis, perform an extensive physical examination for coexisting systemic involvement and secondary causes, and carry out required imaging modalities [1,2,9–11]. Lymphoscintigraphy is the gold standard diagnostic technique with 90% sensitivity and 100% specificity. In recent years, ultrasonography is gaining popularity in lymphedema diagnosis as a noninvasive imaging modality [11].

Pediatric lymphedema may affect the extremities, trunk, genitals, head-face, and rarely the internal organs [6–10], and cause life-long physical, psychological and social problems but is still a neglected condition among physicians [2,6,11–14]. Due to the low awareness about pediatric lymphedema, the patients may be misdiagnosed or late-diagnosed in both developed and developing countries [1,6]. Prevention of progression, early diagnosis, and proper treatment are crucial in the management of pediatric lymphedema. Reducing lymphedema-associated burden and disability in the pediatric setting requires improved awareness and understanding of the development and clinical properties of the lymphedema [4,6,14]. To address these gaps in evidence, we performed a cross-sectional descriptive multi-center study. The aim of this study was to evaluate the clinical characteristics of patients with pediatric lymphedema presented to lymphedema units in different regions of Turkey.

## 2. Materials and methods

The study was approved by the ethics committee of the coordinator university (GO-2018-439/20). The family of the participants provided written informed consent for data collection. An invitation letter was sent to 10 lymphedema centers in different regions of Turkey (registered in Anatolian Lymphedema Association website-www.lenfodemdernegi.org.tr), 8 of them replied but 1 of them could not reach to have least number of patients (n = 5), therefore 7 centers from 4 cities (University of Hacettepe, University of Ege, İstanbul Physical Therapy and Rehabilitation Training and Research Hospital, University of Cumhuriyet, Bakırköy Sadi Konuk Education and Research Hospital, University of Celal Bayar and Ankara Training and Research Hospital) attended to the study.

All lymphedema patients that were referred to lymphedema units of these different hospitals, were screened during April 2018–April 2019, and patients diagnosed with pediatric lymphedema were included in the study. The inclusion criteria were; to be 0–18 years of age and to be diagnosed as lymphedema. Those who are not willing to participate in the study were excluded. Diagnosis of primary and secondary lymphedema was based mainly on anamnesis, physical examination, and lymphoscintigraphic or ultrasonographic evaluation [6,15,16] in which researchers have taken standardized complete anamnesis and performed standardized extensive physical examination [15] in all centers. The main researchers from each center were experienced PMR specialists and members of the Anatolian Lymphedema Association who delivered or received education for standardized diagnosis of lymphedema, depending on consensus documents (6,15,16). The presence of genetic syndromes, comorbid diseases or vascular anomalies were recorded from the files of the patients, in which majority of them were sent from pediatric clinics.

The patient characteristics including age, gender, body mass index (BMI), comorbid diseases, drugs, performed imaging modalities, presence of genetic syndromes, family history, as well as the education level and monthly income of the parents were recorded. Lymphedema characteristics comprising duration of lymphedema, site of lymphedema, limb involvement, stage of lymphedema according to ISL criteria [16], pitting of edema, Stemmer sign positivity (checking the thickness of dermis and fibrosis by lifting the skin on dorsum of fingers [1,15]), complications (infection, cellulitis, papillomatosis, wound, pain) and the received therapies (complete decongestive therapy (CDT), bandaging, manual lymphatic drainage (MLD), skin care, self-management techniques, pneumatic compression pumps, exercise and other therapies like drugs and alternative therapies) for lymphedema were determined. For parents, the education about parental self-management techniques to control swelling and reduce complications was also assessed [14,17].

As gender differences in regard to epidemiology, involvement site, and associated syndromic conditions, may exist in pediatric lymphedema; we additionally analyzed our data according to gender.

### 2.1. Statistical analysis

The analysis of the demographic and clinical data was conducted by descriptive statistics. Normality of continuous variables was evaluated by the Kolmogorov–Smirnov test. All the continuous variables were following normal distribution. Accordingly, continuous data were described (mean + SD) and analyzed using parametric statistics. The categorical variables were recorded as frequencies (n) and percentages (%) and were compared using the Fisher’s exact test. Differences between male and female patients were examined using student’s t tests for continuous and Fisher’s exact test for categorical variables. Pearson (r), Spearman (r_s_) or Eta (r_pb_) correlation coefficient was used to evaluate the correlation between the duration of lymphedema and demographic or clinical parameters. A two-sided p < 0.05 was considered statistically significant. All analyzes were performed with SPSS 22.0 and p < 0.05 was considered significant.

## 3. Results

A total of 122 pediatric lymphedema patients who had referred to the predetermined lymphedema centers during 12 months were recruited to the study. The distribution of the number of patients by centers were as follows: University of Hacettepe: 46 (37.7%); University of Ege: 19 (15.6%); İstanbul Physical Therapy and Rehabilitation Training and Research Hospital: 19 (15.6%); Ankara Training and Research Hospital: 14 (11.4%); Bakırköy Sadi Konuk Education and Research Hospital: 13 (10.6%); University of Celal Bayar: 5 (4%) and University of Cumhuriyet: 5 (4%). The majority of the patients (73%) were sent from outpatient clinics of pediatric departments, while 27% of them were sent from outpatient clinics of Physical Medicine and Rehabilitation departments to the lymphedema units of these centers.

According to demographical properties; the female/male ratio was comparable (66/56) with a median age of 126 (min: 5–max: 160) months. The average duration of edema was 63 months with a median value of 40 (2–216) months. Family history was present in 14.7% of children. Lymphedema was part of a syndrome in 18% of the children and most of the patients had no genetic analysis. Nearly half of them (51%) had lymphoscintigraphic evaluation while 58% of them had US results as a diagnostic imaging modality. The most commonly used drugs were oral or topical antibiotics and antifungal agents. Most of the parents’ education level was high school followed by primary school. The monthly income was low in the majority of the families. Table 1 indicated the socio-demographical properties of the study group.

Regarding the lymphedema properties; the majority of the children (92%) had primary lymphedema, while 8.2% of them had secondary lymphedema. The identified causes in children with secondary lymphedema were mainly trauma (marble fall, vehicle accident), infection (phlebitis during intravenous drug therapy, sepsis) and drugs (sirolimus), as recorded from the files. The most common site of involvement was lower extremity, followed by upper extremity and majority of them had unilateral limb involvement. Comorbid internal organ involvement consisting protein losing enteropathy, gastrointestinal, genitourinary involvement, and lung disease were determined from files of 8% of patients. Children who had genital lymphedema was 11% and male patients were more likely than female patients in which 3% of them had hydrocele surgery. Stemmer sign was positive in 76% of them and most of them had stage 2 lymphedema, with spontaneous irreversible grade. The characteristics of lymphedema are shown in Table 2. Lymphedema was complicated in 17% of children, mainly with a clinical picture of cellulitis, infection, and pain.

Considering the treatments; only 40% of children reported receiving treatment. The most common treatment was compression therapy including bandaging and pressure garments, and MLD, respectively. Surgical operation for lymphedema was performed on 2% of the children, mainly for the lymphedema in the genital area. As with other therapies; 8 children got oral or topical antibiotics and/or antifungal therapies, and 1 of them got leech therapy. No family or children was educated in self-care management before.

There was a statistically significant difference between male and female children only in regard to age (Table 3). Female patients were more likely to be older, but the other variables were similar between gender groups.

The duration of lymphedema was correlated with stage (r_s_: 0.345, p < 0.001) and grade (r_s_: 0.363, p < 0.001) of lymphedema and as well as with Stemmer sign positivity (r_pb_: 0.774, p = 0.03) and BMI (r: 0.291, p: 0.003) (Table 4).

## 4. Discussion

Our study group with 122 children; had a comparable gender distribution and majority of our patients had primary lymphedema usually involving the lower extremities. Lymphedema was part of a syndrome in 18% of the children and complicated in 17% of them, commonly with a clinical picture of infection and pain. Although most of the children had stage-2 lymphedema, only 40% of them received treatment which was mainly the compression therapies. No family or child was educated for self-care management before.

Lymphedema is a rare condition affecting approximately 1.15–4 in 10,000 children and adolescents (5,16) but is frequently misdiagnosed and unrecognized due to the heterogeneous clinical picture and the natural variable course [6,14,18]. There are limited series in the literature defining the clinical characteristics and progressive course of the disease in patients with pediatric lymphedema [3,5,18–20]. As far as we have known, this is the first study that presents the clinical and demographical characteristics of Turkish pediatric lymphedema patients.

The etiology of pediatric lymphedema commonly depends on congenital primary conditions but conditions like trauma, infection, drugs, lipedema, Kawasaki syndrome may also cause secondary lymphedema in children [7,18–22]. The previous series reported that 86% to 93% of the children had primary lymphedema, and secondary causes of pediatric lymphedema were mostly reported as trauma, infection, sepsis, drugs like sirolimus, and sodium valproate. [5,18,19,22]. In our study group, we have determined 92% of children with primary lymphedema and the main reasons for secondary lymphedema were mostly the trauma and infection.

Rarely, the lymphedema can be a part of a systemic syndrome and, pleural and pericardial effusions, ascites, chylous effusions, and pulmonary and intestinal lymphangiectasia can manifest as feature of a more widespread lymphatic problem [10,18,22,23]. Accurate diagnosis is a major concern in pediatric lymphedema as the symptoms may also resemble or misdiagnose with conditions like hemihypertrophy or vascular anomalies [7,22,23]. The association of primary lymphedema with congenital genetic syndromes has been reported with a ratio between 16%–33% in earlier series [4,6,18–20]. Family history of lymphedema is important in congenital primary lymphedema and was determined in 14%–27% of the pediatric patients in the literature [3,5,18–20]. Our data indicated that family history was present in 14% of the children while, congenital genetic anomalies or systemic syndromes were present in 18% of them, as recorded from their files. Most of our patients did not undergo genetic screening for mutations during their assessment in pediatric wards. We did not further analyze the genetic mutations as this point was not the primary aim of our study. Accordingly, the frequency of congenital diseases like Milroy or Meige’s disease may have underestimated in our study group.

Primary pediatric lymphedema is commonly seen in female children and some studies reported the ratio of female pediatric lymphedema as 59%–78% [3,5,20,21–26]. In our study group, the distribution of female and male children with primary lymphedema was comparable with a ratio of 54/46. The extremities, mainly the lower extremity was the most commonly affected site in the literature [3,18,20] but the upper extremity, abdomen, genital area, and trunk were also involved in previous studies [3,5,20]. Schook et al. [19] reported extremity lymphedema in 95% of their patients of which half of them had bilateral and 18% had additional genital swelling. The bilateral involvement was similar between their male and female pediatric patients. Watt et al. [18] indicated 94% of patients with lower limb involvement and 15% of them had genital lymphedema. The ratio of unilateral to bilateral extremity involvement varied from approximately 1:1 to 3:1 in previous reports [4,6,19]. We have determined unilateral/bilateral extremity involvement as 2/1, which was comparable to previous data. The majority of our population had primary lymphedema in the extremities. We have determined 86.8% of lower extremity involvement followed by upper extremity (22.1%) and genital involvement (11.4%).

Lower extremity lymphedema commonly begins from the dorsum of the foot and the edema is usually pitting in early stage. With the progression of the disease, the edema becomes nonpitting and Stemmer sign (unable to pinch the fold of skin on the dorsum of phalanges) can be detected as positive indicating progressed disease [1,2,6]. We recorded pitting in only 38% of our patients and Stemmer sign positivity was %76 in our study group which indicates the fibrosis and advanced stage of the extremity lymphedema. Most of our study participants had stage 2 lymphedema with spontaneous irreversible type. None of the previous studies indicated the stage of their patients but reported the complications of lymphedema that indirectly show the progressed disease.

Lymphedema is a chronic condition and several complications including pain, infection, wounds, lymphorrhea, may develop and lead to physical disabilities [1–3]. A common complication of pediatric lymphedema is the repeated attacks of cellulitis and lymphangitis which was reported in 12%–22% of cases with primary lymphedema [3,5,18–21]. A previous study [18] reported complications of lymphedema in 73% of their patients, most commonly as fibrosis followed by cellulitis and pain. Schook et al. [19] reported cellulitis in 19% of their patients in which recurrent infections were also common. In our study we have determined complications in 17% of children; cellulitis and pain being the most common ones.

Genital lymphedema in pediatric population is common and previous series determined the frequency of genital lymphedema as 8%–18% among children with primary lymphedema [3,19,23,24]. There was a male predominance in the involvement of the genital area in the majority of the studies and most of them had concomitant lower extremity involvement [19,20,24]. The most common complication of genital lymphedema was cellulitis, and the surgical debulking procedures were performed in 36%–44%, resulted in improved symptoms and appearance [3,18,19,24]. We have observed genital lymphedema in 11.7% of our patients, and 64% of them were male. Patients (2%) had surgery with improved clinical picture. Surgical management of genital lymphedema especially in male children is not uncommon [23–25]. A previous study reported that 34 of 56 patients with genital involvement required surgical management, with emphasizing the risk of operation in lymphoedematous tissues and supporting the earlier performance of conservative management [24]. In our study the surgical operations for treatment were performed for only male genital lymphedema.

The diagnosis of lymphedema in children is often delayed and there are difficulties to reach to the proper care and management [6,16,26]. There are several studies that have enlightened the delay for diagnosis and treatment of pediatric lymphedema [2,5,6,13–15,20]. We did not determine the duration of diagnosis as timing was not clear in some of our study group. But according to the clinical examination findings, and the stage of lymphedema; we can comment that most of the children had advanced disease, which may indirectly indicate the delayed diagnosis and/or treatment. The delay in diagnosis and treatment may be due to the relative rarity of the condition, heterogeneous clinical picture, lack of awareness and information about the condition, and lack of available services for the care and management of lymphedema in our region [27].

There is no cure for this lifelong condition, but CDT (skin care, manual lymphatic drainage, multilayer bandaging, exercise, pressure garments, self-care education) as a gold standard of lymphedema treatment, reduce the volume, decrease the incidence of complications and improve quality of life [1,2,13,27,28]. The CDT principles resemble to those for adults but some modifications may be needed in compression degrees and pressure garments. In addition, self-care management techniques are the cornerstone of the therapy and long-term well-being. Pediatric specific management strategies should include teaching parents to take active role in management, encouraging normal physical activity, and the inclusion of psychosocial support among children [1,13,29,30]. During the life-long maintenance phase, compliance and adherence of children to self-management are essential to provide the adequate symptom control. According to our results more than half of the patients did not receive any therapy before, and none of them was educated for self-care management methods. In other words; self-management education was neglected and missed, despite 40% of them received at least one component of CDT. Therefore, health professionals should be educated on the importance and teaching of self-care methods in children with lymphedema. The most common treatment was recorded as compression therapies including bandaging and pressure garments, followed by skin-care and MLD. The majority of pediatric patients in previous retrospective studies were reported to receive compression therapies and MLD, supporting our data [3,5,18,19]. There is no pharmacological therapy for lymphedema that have proven benefit, in contrast some drugs may worsen the disease [29,30]. The used drugs in our study were commonly prescribed for the complications of lymphedema and comorbid vascular anomalies.

Although pediatric lymphedema is rare, the impact on the lives of the children and their families is great. Children growing up with lymphedema can struggle during key developmental stages and have to cope with a physical disability, to struggle with low self-esteem, and social and lifestyle restrictions. This can be even harder for the families who are desperate to understand what is happening to their child, and who are seeking for the true diagnosis, information, and suitable management for their child’s condition [6,14,17,20]. In addition, they have to struggle to manage the financial problems related to the treatments [6,14,17,19]. In our study, most of the families had low monthly income according to the economic status of the country. In Turkey the CDT therapies in government hospitals are free but the materials used in bandaging are not reimbursed. More importantly, the pressure garments that need to be changed every 6 months in the maintenance phase, are reimbursed at about 30% rate. All these points lead to difficulties in reaching the proper treatments and maintaining the improvements in the long-term base. The relatively small study group and the cross-sectional design of the study are the limitations of our study. But as far as we have known this is the first study evaluating the detailed demographic and clinical variables for this population, which may highlight the unmet need for the early diagnosis and proper management of pediatric lymphedema patients in a developing country. The multicenter design may also strengthen our results. We hope the results of our study help to improve the health care delivery settings in order to enhance the quality of life of these patients, as a strategical approach of the government.

In conclusion pediatric lymphedema has a comparable gender distribution and usually involves the lower extremities in our study group. The duration of the disease was long and more than half of patients had spontaneous irreversible lymphedema at submission. Although most of the children had advanced disease, more than half of the patients did not receive any treatment indicating the unmet need for management of lymphedema. The education of the parents and/or children about self-management methods were lacking. We suggest educational activities for both families of children with lymphedema and health care providers, in order to facilitate early reference to lymphedema units and, to receive prompt preventive and therapeutic approaches for this suffering condition.

## Figures and Tables

**Table 1:** The sociodemographical characteristics of the participants.

	Patients (n = 122)	
**Age (month)**	120.74 ± 71.29	
**Gender**		
Female	66 (54%)	
Male	56 (46%)	
**BMI (kg/m** **^2^** **)**	20.60 ± 5.46	
**Duration of lymphedema (month)**	63.97 ± 58.7	
**Education of caregiver**	**Mother**	**Father**
Illiterate	3 (2.5%)	1 (0.8%)
Primary school	52 (43.0%)	28 (23.3%)
High School	50 (41.3%)	59 (49.2%)
University	16 (13.2)	32 (26.7)
**Drugs**		
Absent	91 (74.6%)	
Antibiotics/antimycotics (topical/oral)	24 (19.7%)	
Diosmin hesperidin	3 (2.5%)	
Sirolimus	4 (3.3%)	
**Presence of genetic syndrome**		
Present	22 (15.4%)	
Absent	100 (69.9%)	
Turner syndrome	8 (5.6%)	
Down syndrome	3 (2.1%)	
Digeorge syndrome	1 (0.7%)	
Klippel Trenaunay Syndrome	1 (0.7%)	
Hannekam syndrome	1 (0.7%)	
Milroy disease	4 (2.8%)	
Meige’s disease	3 (2.1%)	
**Family history**		
Present	18 (14.8%)	
Absent	104 (85.2%)	
**Imaging used for diagnosis**		
**Lymphoscintigraphy**		
Absent	60 (49.2%)	
Present	62 (50.8%)	
**Ultrasonography**		
Absent	51 (41.8%)	
Present	71 (58.2%)	
**Monthly income (Turkish lira)**		
<1000	4 (4.8%)	
1000–3000	39 (46.4%)	
3000–5000	30 (35.2%)	
5000–7000	3 (3.6%)	
>7000	8 (9.5%)	

BMI: Body mass index

**Table 2:** The clinical properties of lymphedema in all participants.

	n = 122
**Etiology**	
Primary	112 (91.8)
Secondary	10 (8.2%)
**Site of lymphedema**	
Lower extremity	106 (86.9%)
Upper extremity	27 (22.1%)
Trunk	2 (1.6%)
Genital	14 (11.5%)
Head & face	4 (3.3%)
İnternal organ	8 (6.6%)
**Limb involvement**	
Unilateral	39 (32.0%)
Bilateral	83 (68.0%)
**Internal organ involvement**	
Absent	112 (91.8%)
Respiratory system	3 (2.5%)
Gastrointestinal system	5(4.1%)
Genitourinary system	2 (1.6%)
**Stage of lymphedema**	
Stage 0	5 (4.1%)
Stage 1	42 (34.4%)
Stage 2	70 (57.4%)
Stage 3	5 (4.1%)
**Pitting**	
Positive	46 (37.7%)
Negative	76 (62.3%)
**Stemmer sign**	
Positive	93 (76.2%)
Negative	29 (23.8%)
**Complications**	
Absent	101 (82.8%)
Present	21 (17.2%)
Pain	19 (15.6%)
Cellulitis	17 (13.9%)
Papillomatosus skin changes	3 (2.5%)
Wound	2 (1.6%)
**Treatment**	
None	73 (59.8%)
Skin care	30 (24.6%)
Manual lymphatic drainage	23 (18.9%)
Bandaging	35 (28.7%)
Compression garment	29 (23.8%)
Pump	1 (0.8%)
Exercise	22 (18.0%)
Self-care methods	0 (0.0%)
Surgery	2 (1.6%)
Other	8 (6.6%)

**Table 3:** The difference of clinical and demographical variables between male and female pediatric patients.

	Female (n = 66)	Male (n = 56)	P values
**Age (month)**	139.67 ± 66.59	99.12 ± 70.9	0.002*^1^
**BMI (kg/m** **2** **)**	21.30 ± 5.56	19.80 ± 5.39	0.153^1^
**Involvement site**			0.230^1^
Leg	59	47	
Arm	13	14	
Genital	5	9	
Trunk	2	0	
Head/face	3	1	
Internal organ	3	7	
**Duration of disease (month)**	68.77 ± 57.90	59.24 ± 59.89	0.453^1^
**Stage of lymphedema**			0.800^2^
Stage 0	3	2	
Stage 1	20	22	
Stage 2	40	30	
Stage 3	3	2	
**Grade of lymphedema**			0.626^2^
Reversible	20	23	
Spontaneous irreversible	45	32	
Elephantiasis	1	1	
**Complications**			1.000^2^
Present	2	1	
Absent	10	7	
**Treatment**			0.071^2^
None	33	40	
Skin care	22	8	
Bandaging	22	13	
Manual lymphatic drainage	14	9	
Compression garment	20	9	
Pump	1	0	
Exercise	16	6	
Self-care	0	0	
Other	7	3	
**Monthly income (Turkish lira)**			0.639^2^
<2000	3	2	
2000–3000	23	26	
3000–5000	19	14	
5000–7000	11	5	
>7000	10	8	

BMI: Body mass index,

* p < 0.05, female vs. male group,

1 Student t-test,

2 Fisher’s exact test

**Table 4:** Correlation between the duration of lymphedema and other clinical factors.

	Stage	Grade	Stemmer sign positivity	BMI
**The duration of lymphedema**	r: 0.345^1^	r: 0.363^1^	r: 0.774^2^	r: 0.291^3^
	p: 0.0009	p: 0.0009	p: 0.030	p: 0.003

1 Spearman correlation coefficient

2 Point-biserial correlation coefficient

3 Pearson correlation coefficient
